# Unique Cyclized Thiolopyrrolones from the Marine-Derived *Streptomyces* sp. BTBU20218885

**DOI:** 10.3390/md20030214

**Published:** 2022-03-18

**Authors:** Fuhang Song, Jiansen Hu, Xinwan Zhang, Wei Xu, Jinpeng Yang, Shaoyong Li, Xiuli Xu

**Affiliations:** 1 School of Light Industry, Beijing Technology and Business University, Beijing 100048, China; songfuhang@btbu.edu.cn; 2 Laboratory of RNA Biology, Institute of Biophysics, Chinese Academy of Sciences, Beijing 100101, China; jiansenhu@ibp.ac.cn; 3 School of Ocean Sciences, China University of Geosciences, Beijing 100083, China; zhangxinwan@cugb.edu.cn (X.Z.); xuwei1110@cugb.edu.cn (W.X.); yangjinpeng@cugb.edu.cn (J.Y.); 4 School of Pharmacy, Tianjin Medical University, Tianjin 300070, China; lishaoyong@tmu.edu.cn

**Keywords:** marine-derived *Streptomyces*, thiolopyrrolone, antibacterial, *M. tuberculosis*

## Abstract

Two new cyclized thiolopyrrolone derivatives, namely, thiolopyrrolone A (**1**) and 2,2-dioxidothiolutin (**2**), together with the kn own compound, thiolutin (**3**) were identified from a marine-derived *Streptomyces* sp. BTBU20218885, which was isolated from a mud sample collected from the coastal region of Xiamen, China. Their chemical structures were determined using spectroscopic data, including HRESIMS, 1D and 2D NMR techniques. **1** possessed a unique unsymmetrical sulfur-containing thiolopyrrolone structure. All the compounds were tested for bioactivities against *Staphylococcus aureus*, *Escherichia coli*, Bacille Calmette–Guérin (BCG), *Mycobacterium tuberculosis*, and *Candida albicans*. **1** displayed antibacterial activities against BCG, *M. tuberculosis*, and *S. aureus* with minimum inhibitory concentration (MIC) values of 10, 10, and 100 μg/mL, respectively. Thiolutin (**3**) showed antibacterial activities against *E. coli*, BCG, *M. tuberculosis*, and *S. aureus* with MIC values of 6.25, 0.3125, 0.625, and 3.125 μg/mL, respectively.

## 1. Introduction

Infectious diseases caused by infectious microorganisms continue to threaten human health. Moreover, the development of drug resistance by *Candida albicans*, *Staphylococcus aureus*, *Escherichia coli*, and *Mycobacterium tuberculosi* is becoming more and more serious in hospitals and the community [1,2,3,4]. There is an urgent need to develop new drugs to fight against these pathogens.

*Streptomyces* belongs to actinomycetes which are highly diverse Gram-positive bacteria with high guanine and cytosine content in their DNA. Actinomycetes are well known as an important resource for screening new antibiotics [5], representing 45% of the bioactive secondary metabolites originating from microorganisms [6]. Moreover, *Streptomyces* are the key source of many of the world’s antibiotics in clinics [7,8]. With the detailed investigation on marine microorganisms, marine-derived actinomycetes have proven to be an inexhaustible source for bioactive secondary metabolites [9]. A number of new bioactive compounds were characterized from marine-derived *Streptomyces*, such as isoquinolinequinones [10], terpenoid derivatives [11,12], angucycline derivatives [13,14,15], glycosylated aromatic polyketides [16], bicyclic peptides [17], depsipeptides [18], benzodiazepines [19], and piericidin derivatives [20].

**Figure 1:**
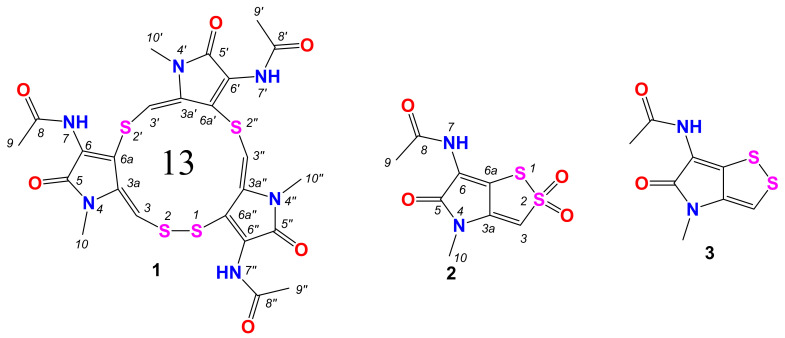
Chemical structures of **1**–**3**.

In the course of our screening of antibacterial secondary metabolites from marine-derived actinomycetes [21,22,23,24], the EtOAc extract of *Streptomyces* sp. BTBU20218885, isolated from a mud sample collected from the coastal area of Xiamen, Fujian Province, China, showed antibacterial activity against Bacille Calmette–Guérin (BCG), the live attenuated vaccine form of *Mycobacterium bovis*, with minimum inhibitory concentration (MIC) of 20 μg/mL. A chemical investigation of this *Streptomyces* strain resulted in the isolation of two new cyclized thiolopyrrolone derivatives, namely, thiolopyrrolone A (**1**) and 2,2-dioxido thiolutin (**2**), together with the known compound, thiolutin (**3**) (Figure 1). Details of fermentation, isolation, structural elucidation, and antibacterial activities are reported here.

## 2. Results

### 2.1. Structure Elucidation

**Figure 2:**
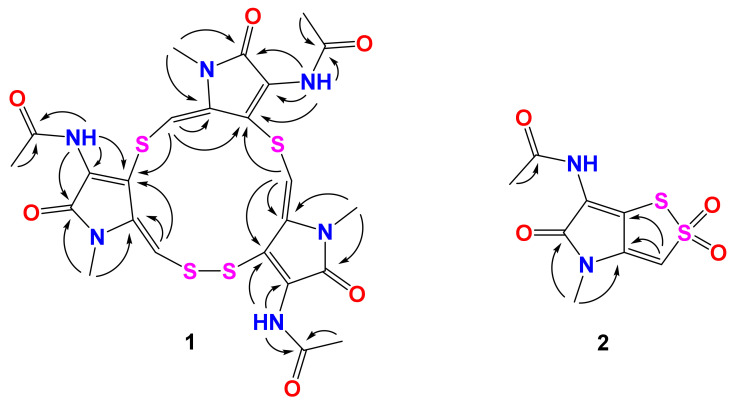
Key HMBC (arrows) correlations in **1** and **2**.

Compound **1** was isolated as a light-yellow amorphous powder. The molecular formula of **1** was deduced to be C_24_H_24_N_6_O_6_S_4_ by the high-resolution electrospray ionization mass spectroscopy (HRESIMS) measurement (*m*/*z* [M+H]^+^ 621.0712, calcd for C_24_H_25_N_6_O_6_S_4_, 621.0713), accounting for sixteen degrees of unsaturation (Figure S1). The ^1^H NMR spectrum (Table 1, Figure S2) showed the presence of three exchangeable singlets (*δ*_H_ 10.27, s, 7-*NH*; 10.21, s, 7′-*NH*; 10.17, s, 7″-*NH*), three olefinic protons (*δ*_H_ 6.58, s, H-3; 6.36, s, H-3′; 6.54, s, H-3″), three *N*-Me groups (*δ*_H_ 3.19, s, Me-10; 3.47, s, Me-10′; 3.40, s, Me-10″), as well as three methyl singlets for acetyl groups (*δ*_H_ 2.07, s, Me-9 and Me-9″; 2.06, s, Me-9′). The ^13^C NMR spectrum (Figure S3), in association with the heteronuclear single quantum correlation (HSQC) spectrum (Figure S4), indicated 24 carbon signals (Table 1), including six methyls (*δ*_C_ 29.9, C-10; *δ*_C_ 29.2, C-10′/C-10″; *δ*_C_ 22.9, C-9; *δ*_C_ 22.8, C-9′/C-9″), three sp^2^ methines (*δ*_C_ 111.9, C-3; *δ*_C_ 112.5, C-3′; *δ*_C_ 112.8, C-3″), six amide carbonyls (*δ*_C_ 164.0/163.7/163.8, C-5/C-5′/C-5″; *δ*_C_ 168.4/168.3/167.9, C-8/C-8′/C-8″), and nine sp^2^ quaternary carbons (*δ*_C_ 137.1/131.9/133.8, 3a/3a′/3a″; *δ*_C_ 130.7/129.8/132.7, 6/6′/6″; *δ*_C_ 124.6/124.6/126.4, 6a/6a′/6a″). The amide carbonyls and olefinic carbons accounted for twelve degrees of unsaturation, which indicated compound **1** was a tetracyclic molecule. Comparison of the NMR data with those of the known compound thiolutin (**3,** Table 1) [25] revealed that **1** was an analogue of thiolutin with a pseudo trimer structure. Furthermore, the heteronuclear multiple bond correlation (HMBC) correlations (Figure 2, Figures S5 and S6) from H_3_-9 and H-7-*NH* to C-8, H_3_-9′ and H-7′-*NH* to C-8′, and H_3_-9″ and H-7″-*NH* to C-8″ revealed the presence of three acetamides. The HMBC correlations from H_3_-10 to C-3a and C-5, H_3_-10′ to C-3a′ and C-5′, H_3_-10″ to C-3a″ and C-5″ confirmed the presence of three *N*-Me amides. The moieties of 1,5-dihydro-2*H*-pyrrol-2-one were determined by HMBC correlations from H-3 to C-3a and C-6a, H-7-*NH* to C-6 and C-6a, H-3′ to C-3a′ and C-6a′, H-7′-*NH* to C-6′ and C-6a′, H-3″ to C-3a″ and C-6a″, H-7″-*NH* to C-6″ and C-6a″. The monosulfur bonds between C-6a and C-3′, C-6a′ to C-3″ were revealed by the HMBC correlations from H-3′ to C-6a, and H-3″ to C-6a′. The absence of HMBC correlation from H-3 to C-6a″, together with molecular formula of **1**, indicated that the C-6a and C-3 were linked through a disulfur bond. Thus, the structure of **1** was established (Figure 1) and named thiolopyrrolone A.

**Figure 3:**
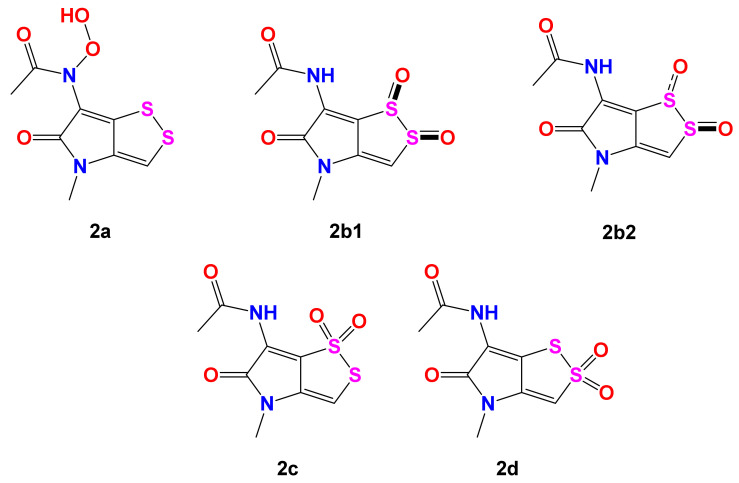
Four possible structures of **2** for calculating ^13^C NMR data in DMSO-*d*_6_.

**Figure 4:**
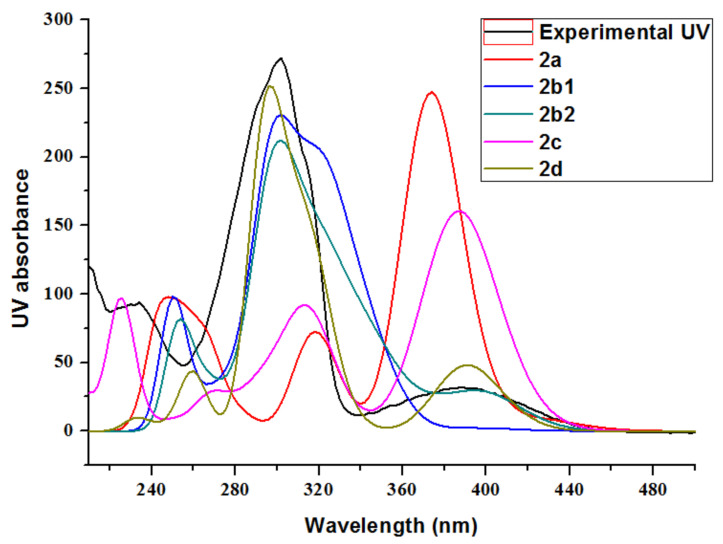
Calculated UV spectra for **2a**–**2d** and UV spectrum for compound **2**.

Compound **2** was isolated as a yellow amorphous powder. The molecular formula of **2** was deduced to be C_8_H_8_N_2_O_4_S_2_ by the HRESIMS measurement (*m*/*z* [M + H]^+^ 260.9996, calcd for C_8_H_9_N_2_O_4_S_2_, 260.9998), accounting for six degrees of unsaturation (Figure S7). The ^1^H ^13^C and HSQC spectra (Figures S8–S10) displayed similar signals to those of **3**, including one sp^2^ methine (*δ*_H_ 7.56, s, H-3; *δ*_C_ 109.6, C-3), one *N*-Me group (*δ*_H_ 3.10, s, H_3_-10; *δ*_C_ 27.9, C-10), and one methyl singlet for acetyl group (*δ*_H_ 2.10, s, H_3_-9; *δ*_C_ 22.6, C-9), two amide carbonyls (*δ*_C_ 164.3, C-5; *δ*_C_ 170.5, C-8), and three sp^2^ quaternary carbons (*δ*_C_ 145.5, C-3; *δ*_C_ 114.1, C-6, *δ*_C_ 123.1, C-6a). The HMBC spectrum (Figure S11) showed correlations from H_3_-9 to C-8, H_3_-10 to C-3a and C-5, H-3 to C-3a and C-6a (Figure 2). Together with the molecular formula calculated by HRESIMS, there are two more oxygen atoms in compound **2**. So there are four possible structures for **2** as shown in Figure 3. The two oxygen atoms for sulfoxides formed *cis* and *trans* conformations, but the optical rotation did not reveal any solid data because of the decomposition of **2**. So, both of the conformations were subjected to quantum chemical calculation. By comparing the experimental and calculated ultraviolet spectra of **2a**–**2d** (Figure 4), the structures of **2b2** and **2d** are consistent with those of experimental data. In order to confirm the structure of **2**, the ^13^C NMR data of the four possible structures were also calculated by density functional theory (DFT). The data were evaluated based on the statistical parameters including correlation coefficient (*R*^2^) between experimental and calculated ^13^C NMR spectroscopic data with a linear regression, the maximum error (MaxErr), and mean absolute error (MAE). Comparison of all these parameters for calculated ^13^C chemical shifts of the four possible isomers with experimental data revealed the best fit was **2d** (Table 2). Thus, the structure of **2** was determined and named 2,2-dioxidothiolutin.

Compound **3** was isolated as a yellow amorphous powder. The molecular formula of **3** was deduced to be C_8_H_8_N_2_O_2_S_2_ by the HRESIMS measurement (*m*/*z* [M + H]^+^ 229.0100, calcd for C_8_H_9_N_2_O_2_S_2_, 229.0100), accounting for six degrees of unsaturation (Figure S12). The ^1^H ^13^C and HSQC spectra (Figures S13–S15) displayed almost the same signals as those of reported thiolutin **3 [21]**. In the HMBC spectrum (Figure S16), the correlations from H_3_-10 (*δ*_H_ 3.25) to C-3a (*δ*_C_ 136.0) and C-5 (*δ*_C_ 166.1), H-3 to C-3a and C-6a (*δ*_C_ 132.4), H-7-*NH* to C-6 (*δ*_C_ 114.8) and C-6a determined the chemical shifts of C-3, C-6, and C-6a. Thus, the chemical shifts for C-3a and C-6a should be swapped [21].

### 2.2. Biological Activity

These compounds were evaluated for their antimicrobial activities against *C. albicans* ATCC 10231, *S. aureus* ATCC 25923, *Mycobacterium bovis* (BCG Pasteur 1173P2), *M. tuberculosis* H37Rv (ATCC27294), and *E. coli* ATCC 25923. Compound **1** exhibited antibacterial activities against BCG, *M. tuberculosis*, and *S. aureus* with MIC values of 10, 10, and 100 μg/mL, respectively. Thiolutin (**3**) showed antibacterial activities against *E. coli*, BCG, *M. tuberculosis*, and *S. aureus* with MIC values of 6.25, 0.3125, 0.625, and 3.125 μg/mL, respectively (Table 3).

## 3. Materials and Methods

### 3.1. General Experimental Procedures

NMR spectra were obtained on a Bruker Avance 500 spectrometer with residual solvent peaks as references (DMSO-*d*_6_: *δ*_H_ 2.50, *δ*_C_ 39.52). High-resolution ESIMS measurements were obtained on an Accurate-Mass-Q-TOF LC/MS 6520 instrument (Santa Clara, CA, USA) in the positive ion mode. HPLC was performed using an Agilent 1200 Series separation module equipped with an Agilent 1200 Series diode array, Agilent 1200 Series fraction collector, and Agilent ZORBAX SB-C18 column (250 × 9.4 mm, 5 µm).

### 3.2. Microbial Material, Fermentation, Extraction, and Purification

Strain *Streptomyces sp.* BTBU20218885 was isolated from a mud sample collected from the intertidal zone, Xiamen, China, and grown on an ISP2 (yeast extract 0.4%, malt extract 1%, dextrose 0.4%, agar 2%; pH 7.2) agar plate at 28 °C. Colony characteristics of BTBU20218885 are shown in Figure S17. The genomic DNA of BTBU20218885 was extracted using a TINAamp Bacteria DNA Kit. PCR amplification of 16S rDNA was carried out by using universal primers (27f:5′-GAGAGTTTGATCCTGGCTCAG-3′; 1492r: 5′-CTACGGCTACCTTGTTACGA-3′). PCR amplification of the 16S rDNA was performed on TaKaRa PCR Thermal Cycler with the initial denaturation at 94 °C for 5 min, 30 cycles of denaturation (94 °C, 1 min), annealing (55 °C, 1 min), and elongation (72 °C, 1 min 15 s), and a final elongation at 72 °C for 10 min, in a 25 μL system (0.4 μL 20 μM of each primer, 2.5 μL 10× buffer, 2.5 μL 2.5 nM dNTP, 2 U rTap polymerase, and 1 μL DNA template). BTBU20218885 was identified as *Streptomyces* sp. by comparing the 16S rDNA sequence with the GenBank database using the BLAST program. A neighbor-joining (NJ) tree (Figure S18) was constructed using the software package Mega version 5 [26]. The strain was assigned the accession number BTBU20218885 in the culture collection at Beijing Technology and Business University, Beijing. The strain BTBU20218885 was inoculated on an ISP2 agar plate and cultured for 7 days. A 250 mL Erlenmeyer flask containing 40 mL of ISP2 medium was inoculated with BTBU20218885 and incubated at 28 °C (160 rpm) for 36 h. Aliquots (9 mL) of the seed cultures were aseptically transferred to 20 × 1 L Erlenmeyer flasks, each containing 300 mL of MPG media (glucose 1.0%, millet meal 2.0%, cotton seed gluten meal 2.0%, and MOPS 2.0%; pH 7.0), and the flasks were incubated at 28 °C, 160 rpm for 7 days. The culture broths were combined and centrifuged to yield a supernatant and a mycelial cake. The supernatant was extracted by equal volume of ethyl acetate (EtOAc, ×3 times), and the combined EtOAc extracts were evaporated to dryness in vacuo to give a dark residue. The residue was sequentially triturated with hexane, CH_2_Cl_2_, and MeOH to afford, after concentration in vacuo, hexane, CH_2_Cl_2_, and MeOH soluble fractions and precipitate. The precipitate was further purified by HPLC (Agilent ZORBAX SB-C18, 250 × 9.4 mm, 5 μm column, 3.0 mL/min, elution with 30% to 100% acetonitrile/H_2_O (0–20 min) to yield **1** (3.5 mg), **3** (2.6 mg), and **2** (13.2 mg).

Thiolopyrrolone A (**1**): Light-yellow amorphous powder; ^1^H and ^13^C NMR data, Table 1; HRESIMS *m*/*z* 621.0712 [M + H]^+^ (calcd for C_24_H_25_N_6_O_6_S_4_, 621.0713).

2,2-Dioxidothiolutin (**2**): Yellow amorphous powder; ^1^H and ^13^C NMR data, Table 1; HRESIMS *m*/*z* 260.9996 [M + H]^+^ (calcd for C_8_H_9_N_2_O_4_S_2_, 260.9998).

### 3.3. Biological Activity

Compounds **1**–**3** were evaluated for their antimicrobial activities in 96-well plates according to the Antimicrobial Susceptibility Testing Standards outlined by the Clinical and Laboratory Standards Institute Document M07-A7 (CLSI) and our previous report [27,28,29]. The MIC was defined as the minimum concentration of the compound that prevented visible growth of the microbes.

### 3.4. Computational Methods

A random conformational search of starting geometries in Discovery studio 4.0 was used to produce low-energy conformers within a 10 kcal/mol energy, which were subsequently optimized using the DFT method at mPW1PW91/6-31g(2d,p) level with GAUSSIAN 09 [30]. The optimized conformers were further checked by frequency calculation at the same level of theory, and resulted in no imaginary frequencies. The time-dependent density functional theory (TDDFT) calculations of their low-energy conformations within 0–2.5 kcal/mol were performed to simulate their UV–vis spectra at the same level. Similarly, their ^13^C NMR calculations were also carried out by GIAO method at the same level [31]. Solvent effect of dimethylsulfoxide was taken into account in the above calculations by using the polarizable continuum model (PCM).

Their theoretical UV–vis spectra based on Boltzmann statistics were generated in the program SpecDis 1.63 [32] by applying Gaussian band shape with a 0.40 eV exponential half-width from dipole-length rotational strengths. Statistical parameters were used to quantify the agreement between experimental and calculated data, including the correlation coefficient (*R*^2^) between experimental and calculated ^13^C NMR spectroscopic data with a linear regression, the mean absolute error (MAE), and the maximum error (MaxErr) [33]. The correlation coefficient (*R*^2^) was determined from a plot of *δ*_calc_ (*x* axis) against *δ*_exp_ (*y* axis) for each particular compound. The mean absolute error (MAE) was defined as $\frac{1}{n}\sum_{i=1}^{n}|\delta_{\mathrm{calc},\;i}-\delta_{\exp,\;i}|$. The maximum error (MaxErr) was defined as max|*δ*_calc_ − *δ*_exp_|.

## 4. Conclusions

In summary, chemical studies on the marine-derived *Streptomyces* sp. BTBU20218885 resulted in the characterization of three cyclized thiolopyrrolones, including a unique unsymmetrical thiolopyrrolone (**1**), 2,2-dioxidothiolutin (**2**), and the previously reported thiolutin (**3**). Dithiolopyrrolones are a class of structurally intriguing natural products with broad antibacterial spectrum [34]. Most of the analogues are characterized by a unique bicyclic pyrrolinonodithiole, with the differences in the substitution groups on *N*-4 and *N*-7 positions of the holothin core [35,36]; however, thiolopyrrolone A is the first sample of analogues with a macrocyclic skeleton. Compound **1** exhibited antibacterial activities against BCG, *M. tuberculosis*, and *S. aureus* with MIC values of 10, 10, and 100 μg/mL, respectively. Thiolutin (**3**) displayed potential antibacterial activities against *E. coli*, BCG, *M. tuberculosis*, and *S. aureus* with MIC values of 6.25, 0.3125, 0.625, and 3.125 μg/mL, respectively.

## Figures and Tables

**Table 1:** ^1^H (500 MHz) and ^13^C NMR (125 MHz) data of **1**–**3** (DMSO-*d*_6_).

Position	1		2		3	
	*δ*_C_	*δ*_H_ (*J* in Hz)	*δ*_C_	*δ*_H_ (*J* in Hz)	*δ*_C_	*δ*_H_ (*J* in Hz)
3/3′/3″	111.9/112.5/112.8	6.58/6.36/6.54, s	109.6	7.56, s	111.0	7.34, s
3a/3a′/3a″	137.1/131.9/133.8		145.5		136.0	
5/5′/5″	164.0/163.7/163.8		164.3		166.1	
6/6′/6″	130.7/129.8/132.7		114.1		114.8	
6a/6a′/6a″	124.6/124.6/126.4		123.1		132.4	
8/8′/8″	168.4/168.3/167.9		170.5		168.8	
9/9′/9″	22.9/22.8/22.8	2.07/2.06/2.07, s	22.6	2.10, s	22.4	2.02, s
10/10′/10″	29.9/29.2/29.2	3.19/3.47/3.40, s	27.9	3.10, s	27.5	3.25, s
7/7′/7″-NH		10.27/10.21/10.17, s		-		9.99, s

**Table 2:** Comparison of calculated (TMS as a reference standard) and experimental ^13^C data for **2**.

Position	2a	2b1	2b2	2c	2d	2
3	115.2	110.2	111.8	107.4	103.9	109.6
3a	129.4	147.5	140.2	121.2	140.2	145.5
5	162.1	160.5	159.0	159.6	159.2	164.3
6	112.9	130.4	127.9	131.1	121.1	114.1
6a	138.1	135.9	131.3	124.5	129.5	123.1
8	167.3	164.1	163.5	163.4	163.9	170.5
9	21.3	23.1	22.8	23.0	22.6	22.6
10	27.6	27.4	26.8	26.4	27.3	27.9
*R* ^2^	0.9723	0.9776	0.9812	0.9534	0.9898	
MAE	5.6	5.4	5.4	7.3	4.6	
MaxErr	16.1	16.3	13.8	24.3	7.0	

**Table 3:** Antibacterial activities of compounds **1**–**3** (MIC, μg/mL).

Number	*C. albicans*	*S. aureus*	BCG	*M. tuberculosis*	*E. coli*
1	>200	100	10	10	>100
2	>200	50	-	-	>200
3	>200	3.125	0.3125	0.625	6.25
Control	1 ^a^	1 ^b^	0.05 ^c^	0.025 ^c^	1 ^d^

^a^ Rapamycin, ^b^ vancomycin, ^c^ isoniazid, ^d^ ciprofloxacin.

## Data Availability

Data are contained within the text.

## Supplementary Materials

The following are available online at https://www.mdpi.com/article/10.3390/md20030214/s1: Figures S1–S16: HRESIMS, 1D, and 2D NMR for compounds **1**–**3**; Figure S17: Colony characteristics of BTBU20211089; Figure S18: Neighbor-joining phylogenetic tree of strain BTBU20211089.
