# Supplementary material for: Unique Cyclized Thiolopyrrolones from the Marine-Derived Streptomyces sp. BTBU20218885
Source: Mar Drugs. 2022 Mar 18;20(3):214. doi: 10.3390/md20030214 (PMC8953990; doi:10.3390/md20030214)
Supplement: Supplementary file 1 [file marinedrugs-20-00214-s001.zip › marinedrugs-1618619-supplementary.pdf]

SUPPLEMENTARY MATERIAL

Unique Cyclized Thiolopyrrolones from the Marine-Derived *Streptomyces* sp. BTBU20218885

Fuhang Song [1,†], Jiansen Hu [2,†], Xinwan Zhang [3], Wei Xu [3], Jinpeng Yang [3], Shaoyong Li [4] and Xiuli Xu [3,*]

[1] School of Light Industry, Beijing Technology and Business University, Beijing, 100048, P. R. China; (songfuhang@btbu.edu.cn)

[2] Laboratory of RNA Biology, Institute of Biophysics, Chinese Academy of Science, Beijing, 100101, P. R. China(jiansenhu@ibp.ac.cn)

[3] School of Ocean Sciences, China University of Geosciences, Beijing, 100083, P. R. China; zhangxinwan@cugb.edu.cn (X.Z); xuwei1110@cugb.edu.cn (W.X.); yangjinpeng@cugb.edu.cn (J.Y)

[4] School of Pharmacy, Tianjin Medical University, 300070 Tianjin, P. R. China; (lishaoyong@tmu.edu.cn)

† These authors contributed equally to this work

* Correspondence: xuxl@cugb.edu.cn

Table of Contents

Figure S1. HRESIMS spectrum for **1** 3
Figure S2. ^{1}H NMR spectrum (500 MHz, DMSO-d_6) of **1** 3
Figure S3. ^{13}C NMR spectrum (125 MHz, DMSO-d_6) of **1** 4
Figure S4. HSQC spectrum (500 MHz, DMSO-d_6) of **1** 4
Figure S5. HMBC spectrum (500 MHz, DMSO-d_6) of **1** 5
Figure S6. Expanded HMBC spectrum (500 MHz, DMSO-d_6) of **1** 5
Figure S7. HRESIMS spectrum for **2** 6
Figure S8. ^{1}H NMR spectrum (500 MHz, DMSO-d_6) of **2** 6
Figure S9. ^{13}C NMR spectrum (125 MHz, DMSO-d_6) of **2** 7
Figure S10. HSQC spectrum (500 MHz, DMSO-d_6) of **2** 7
Figure S11. HMBC spectrum (500MHz, DMSO-d_6) of **2** 8
Figure S12. HRESIMS spectrum for **3** 8
Figure S13. ^{1}H NMR spectrum (500 MHz, DMSO-d_6) of **3** 9
Figure S14. ^{13}C NMR spectrum (125 MHz, DMSO-d_6) of **3** 9
Figure S15. HSQC spectrum (500MHz, DMSO-d_6) of **3** 10
Figure S16. HMBC spectrum (500MHz, DMSO-d_6) of **3** 10
Figure S17. Colony characteristics of BTBU20218885 11
Figure S18. Neighbor-joining phylogenetic tree of BTBU20218885 11

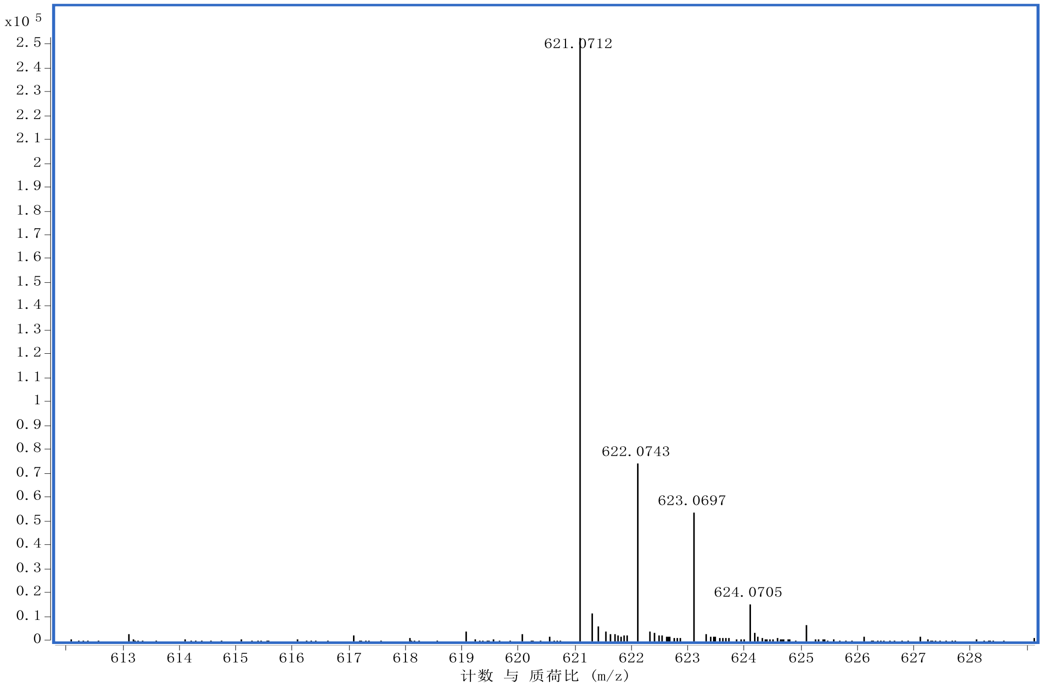

Figure S1. HRESIMS spectrum for compound **1**

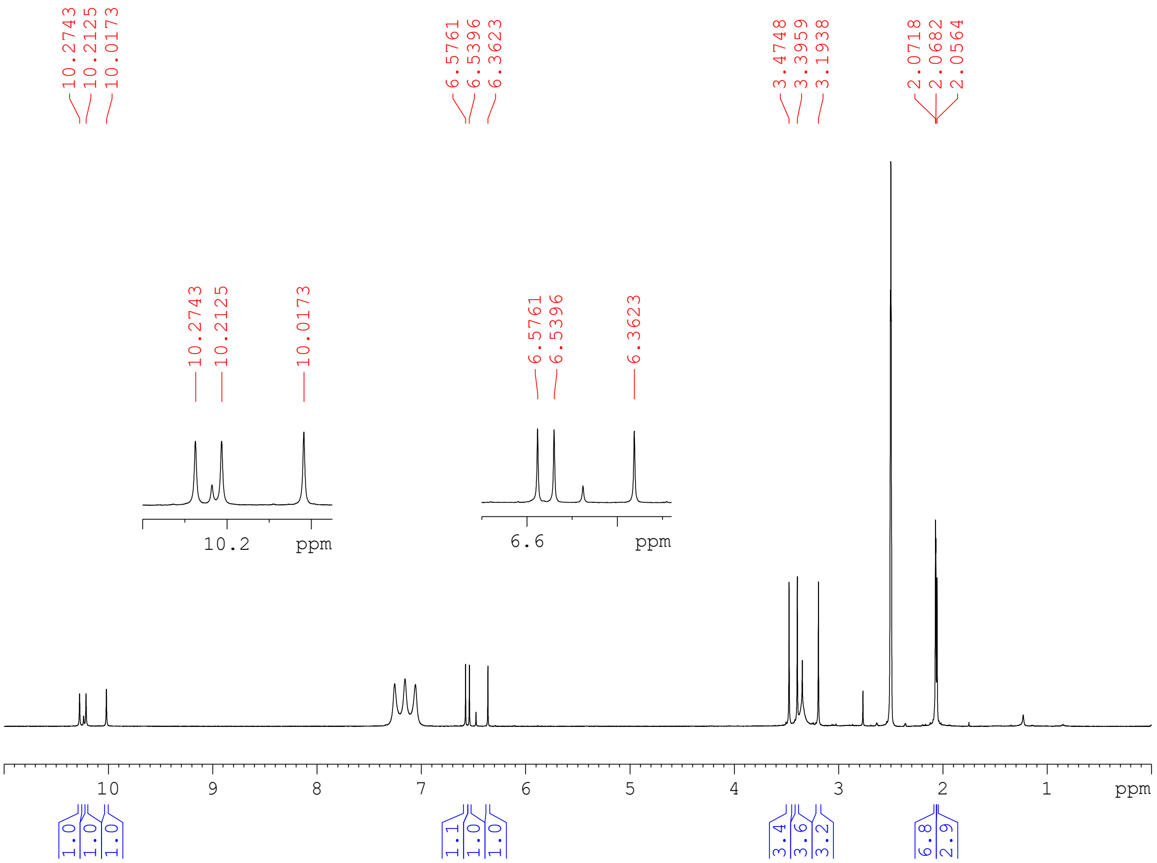

Figure S2. ^{1}H NMR spectrum (500 MHz, DMSO-d_6) of **1**

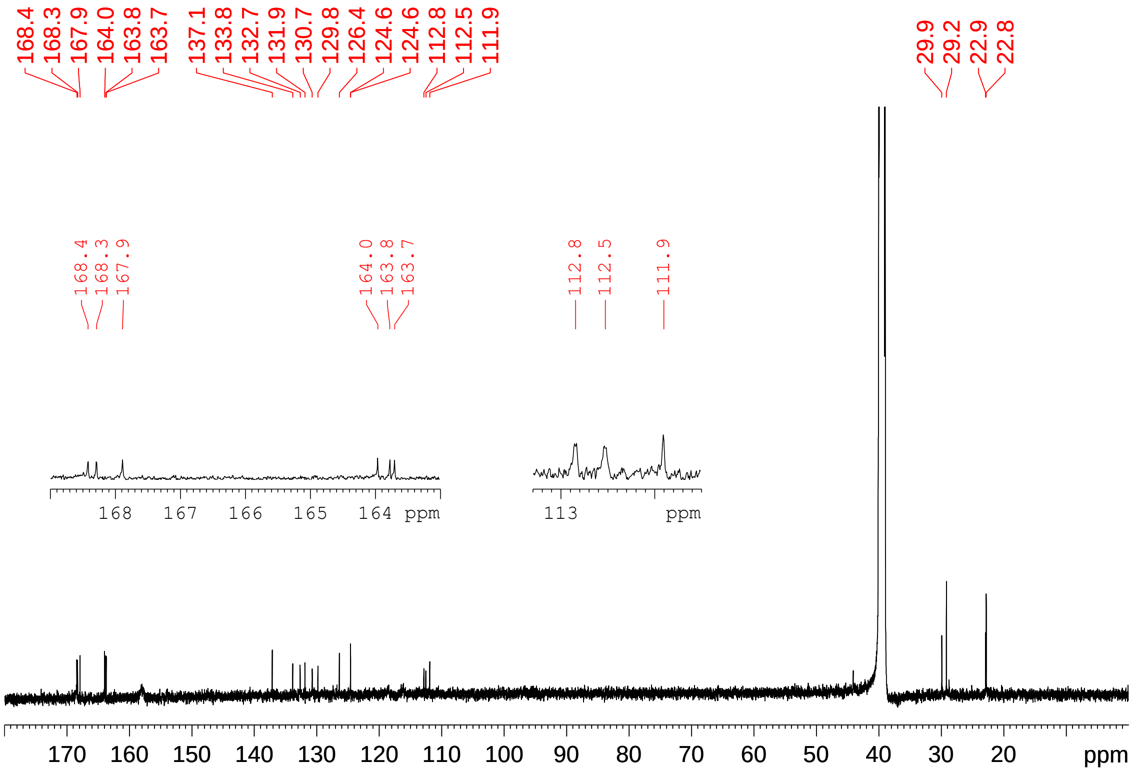

Figure S3. ^{13}C NMR spectrum (125 MHz, DMSO-d_6) of **1**

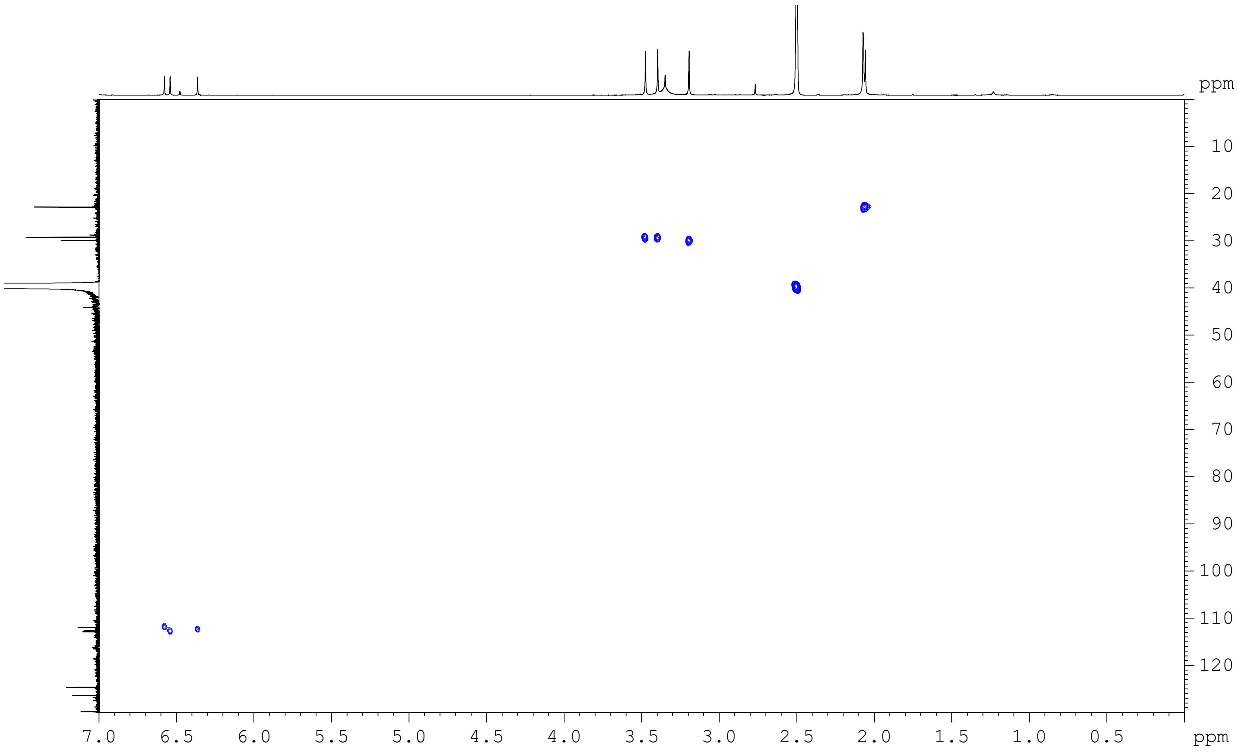

Figure S4. HSQC spectrum (500 MHz, DMSO-d_6) of **1**

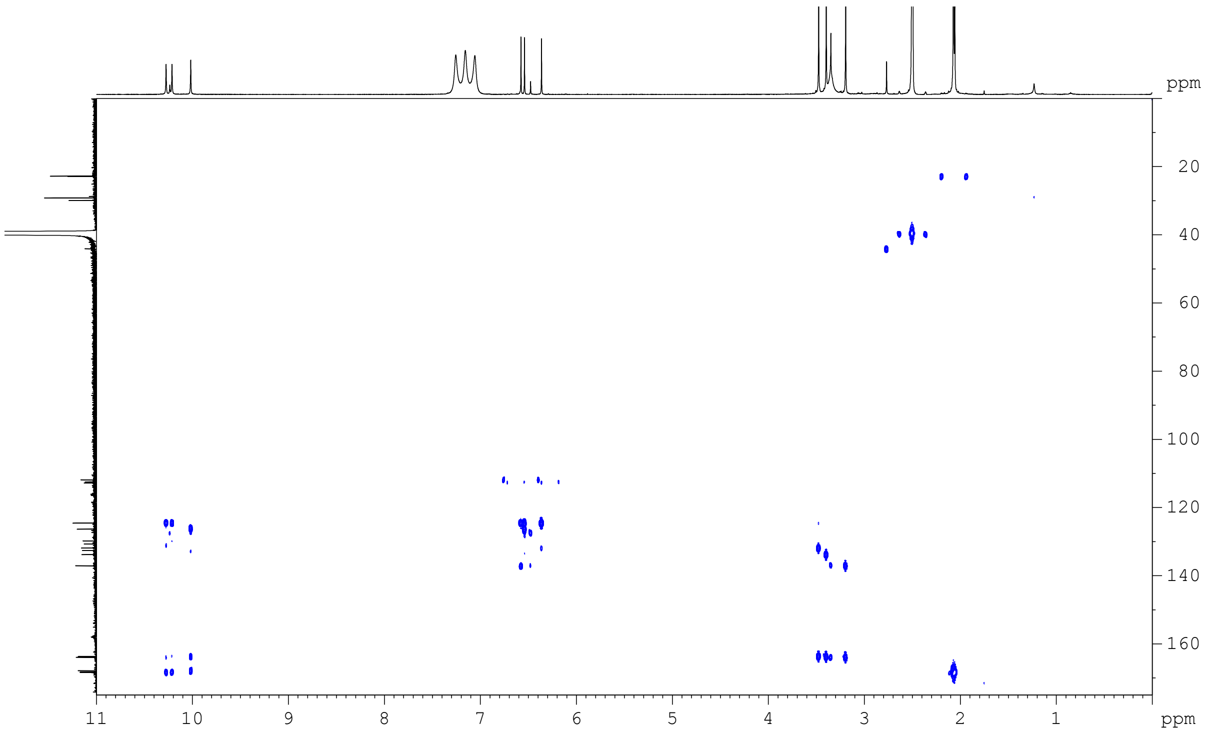

Figure S5. HMBC spectrum (500 MHz, DMSO-d_6) of **1**

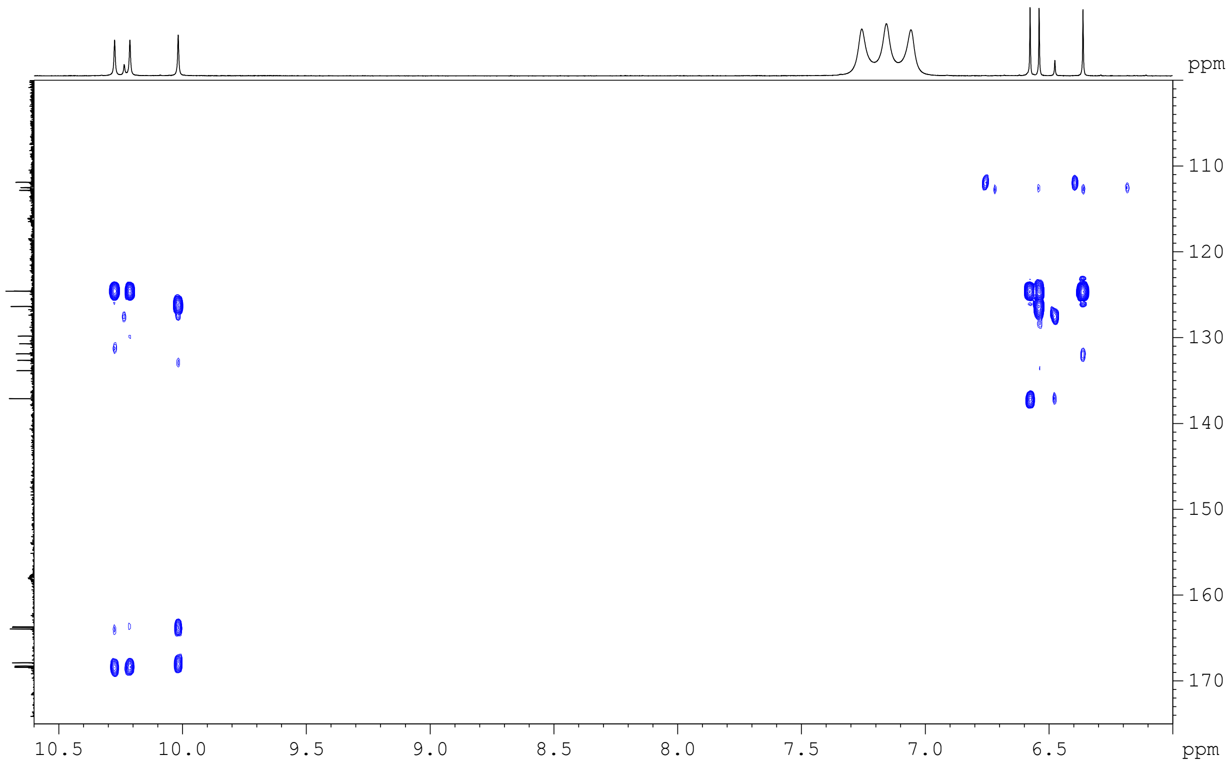

Figure S6. Expanded HMBC spectrum (500 MHz, DMSO-d_6) of **1**

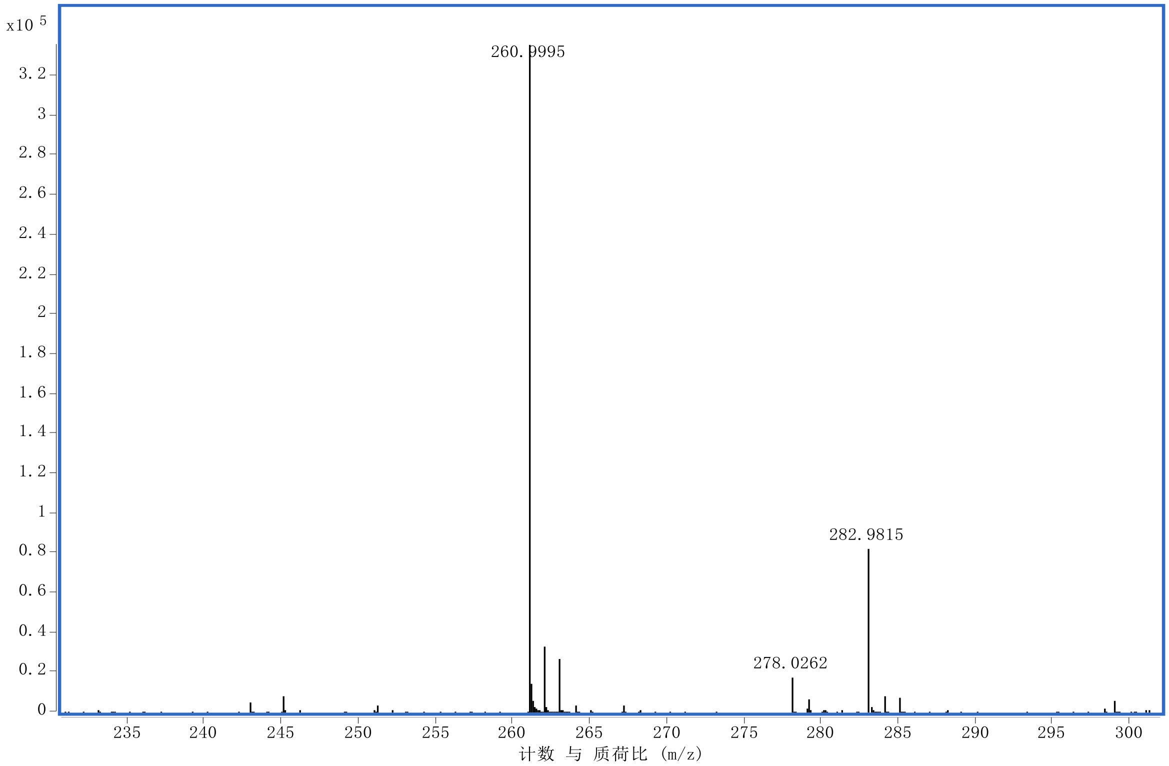

Figure S7. HRESIMS spectrum for **2**

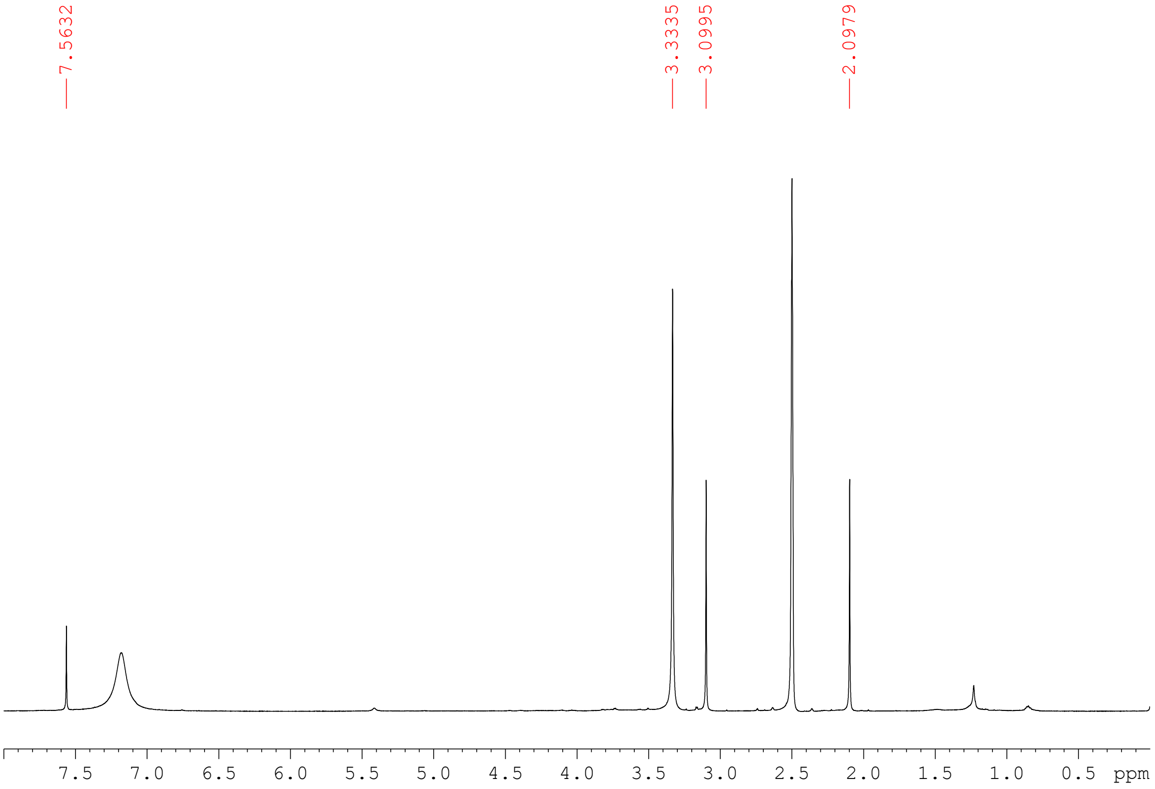

Figure S8. ^{1}H NMR spectrum (500 MHz, DMSO-d_6) of **2**

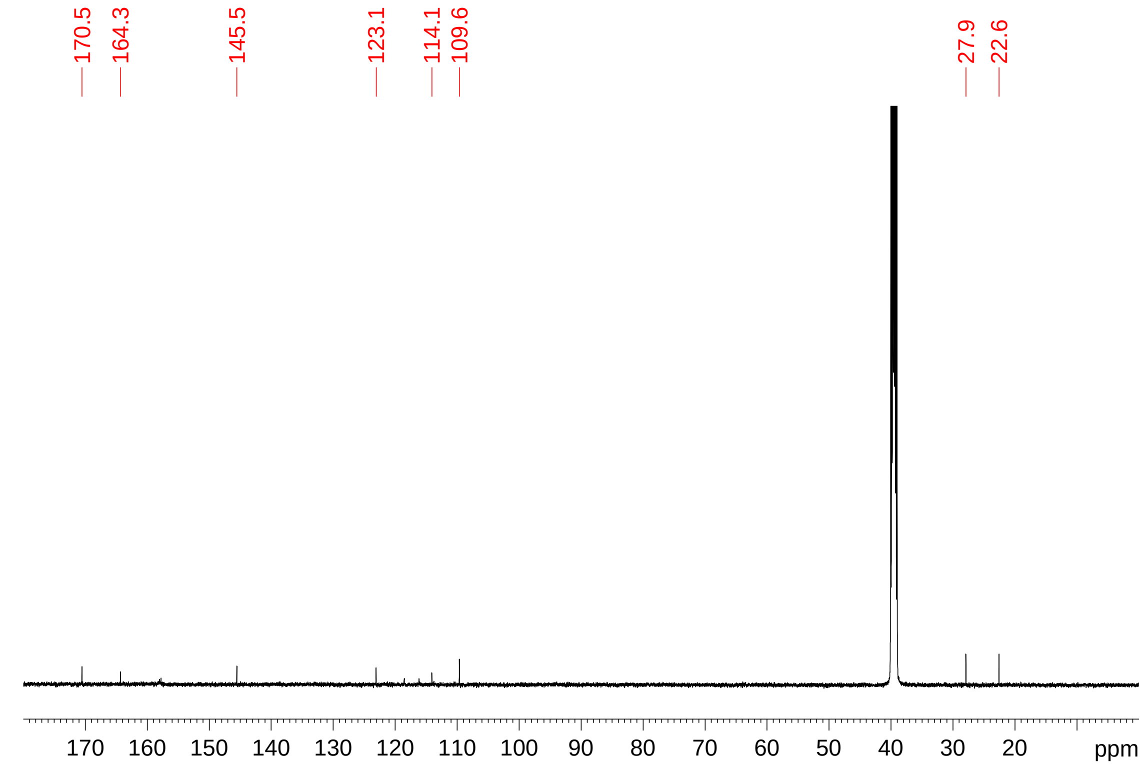

Figure S9. ^{13}C NMR spectrum (125 MHz, DMSO-d_6) of **2**

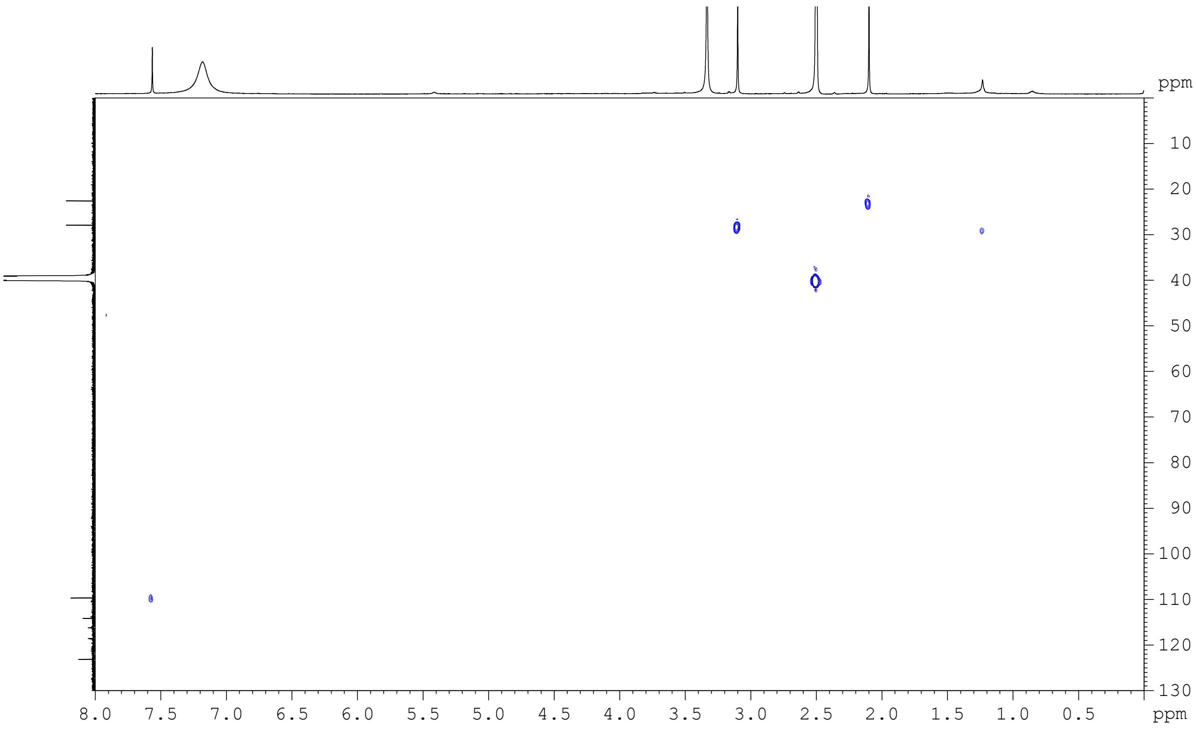

Figure S10. HSQC spectrum (500 MHz, DMSO-d_6) of **2**

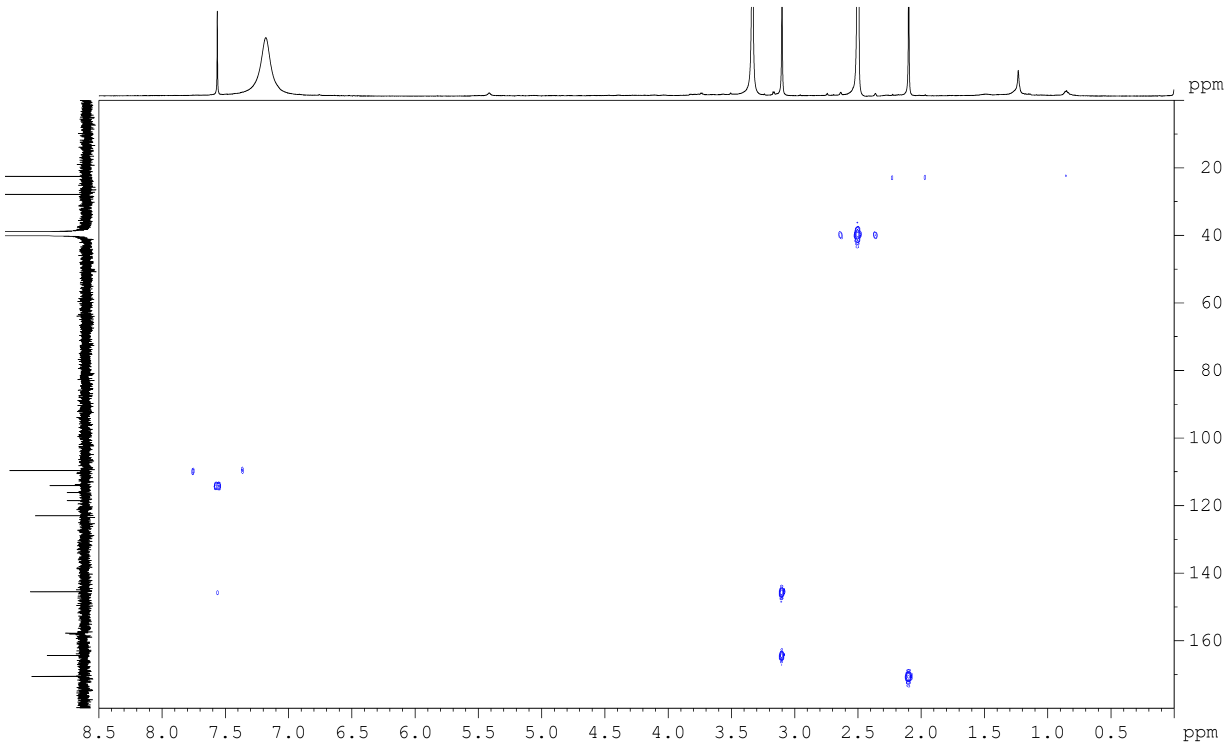

Figure S11. HMBC spectrum (500 MHz, DMSO-d_6) of **2**

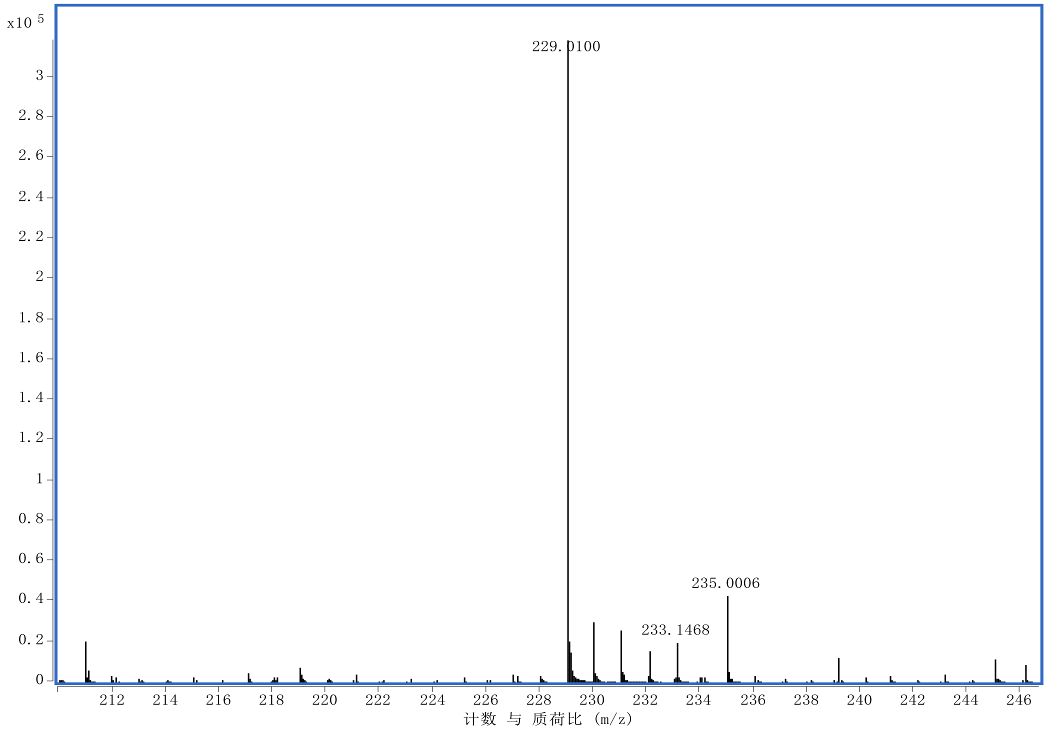

Figure S12. HRESIMS spectrum for **3**

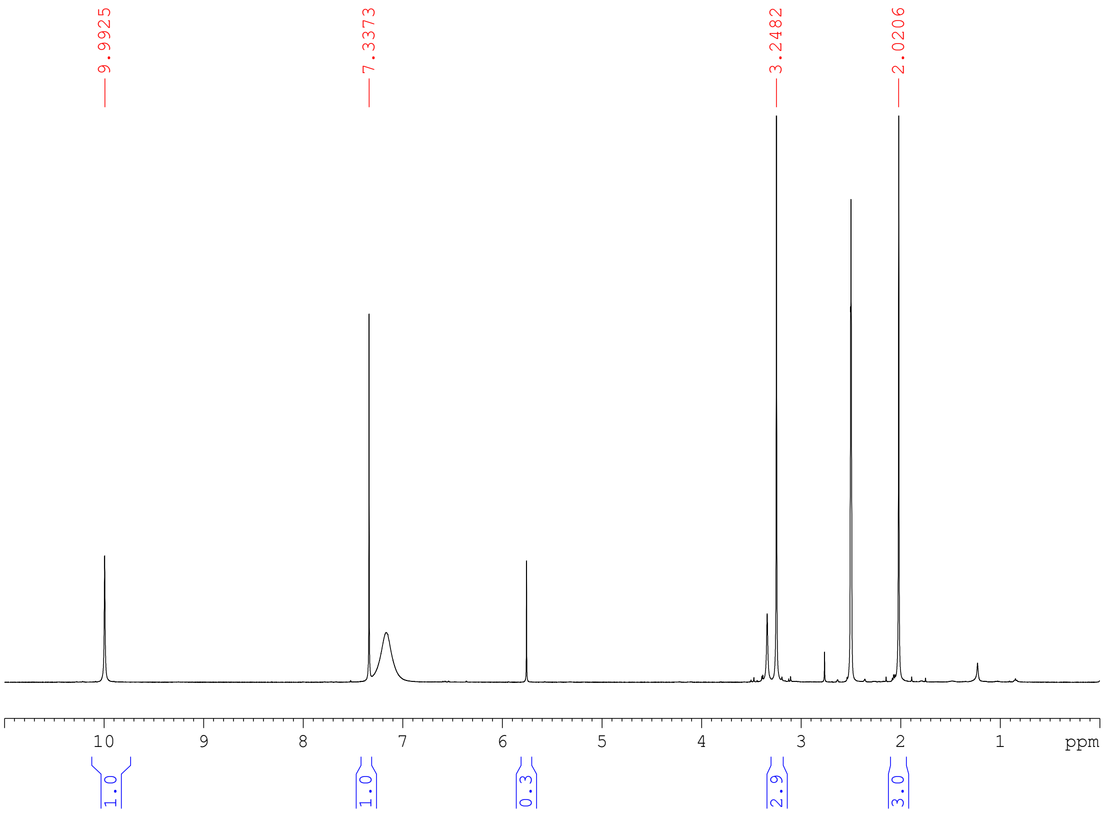

Figure S13. ^{1}H NMR spectrum (500 MHz, DMSO-d_6) of **3**

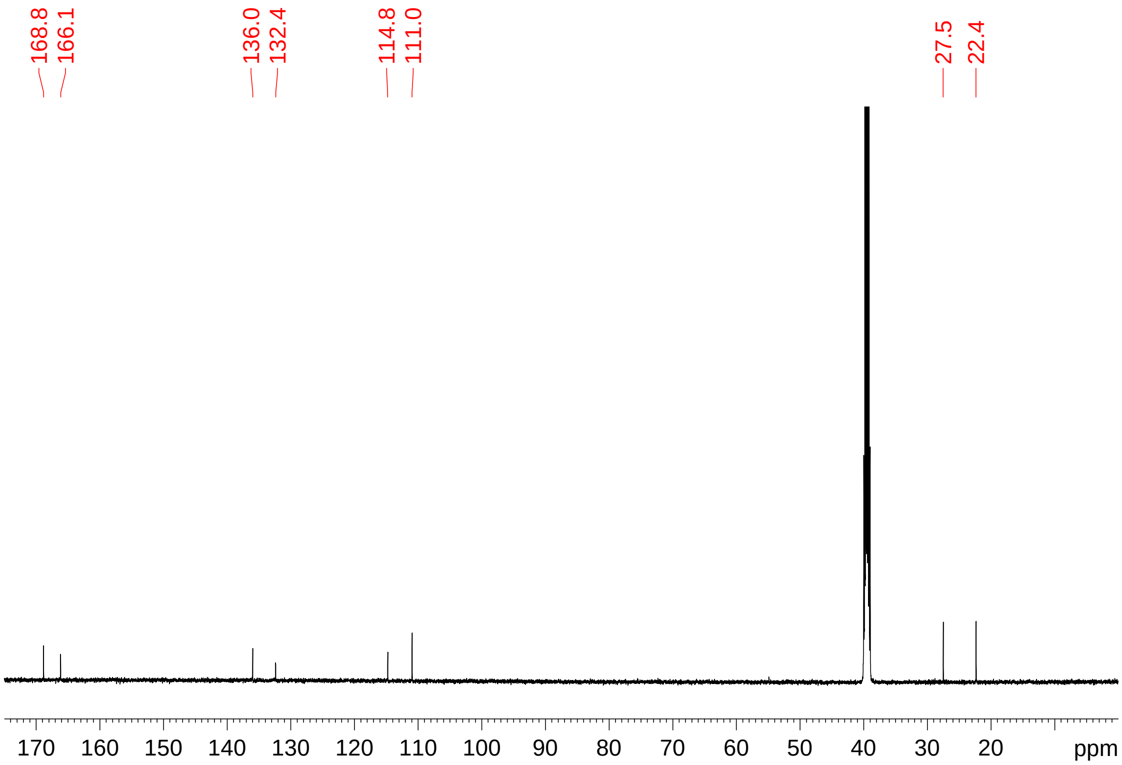

Figure S14. ^{13}C NMR spectrum (125 MHz, DMSO-d_6) of **4**

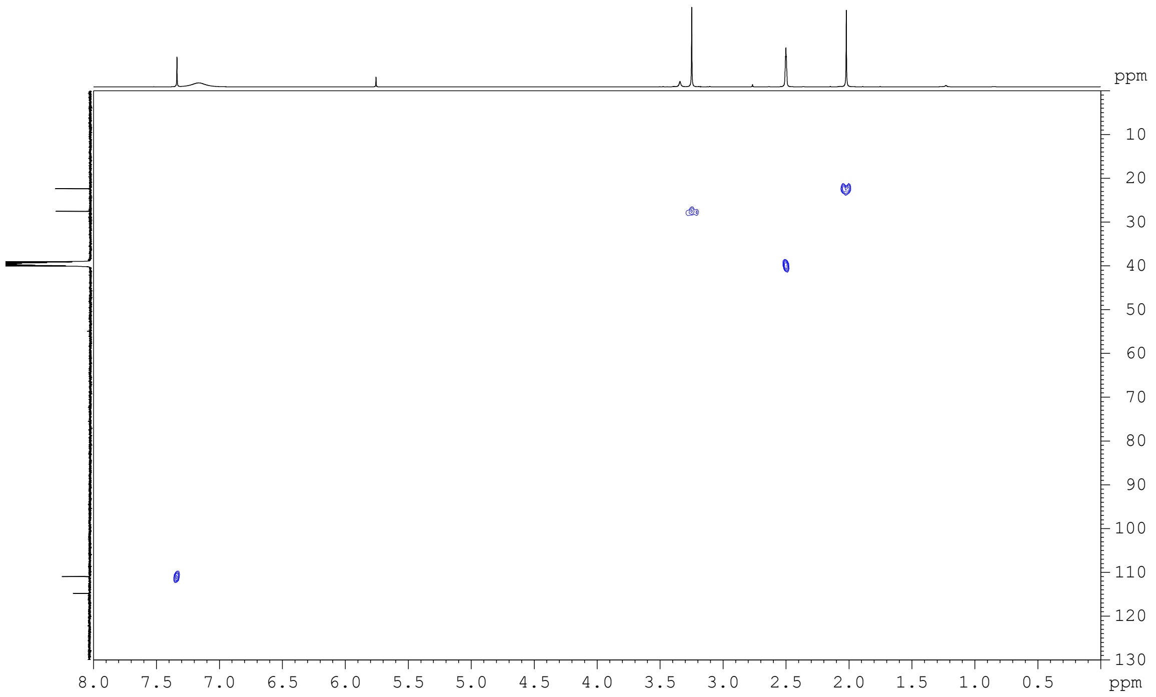

Figure S15. HSQC spectrum (500 MHz, DMSO-d_6) of **4**

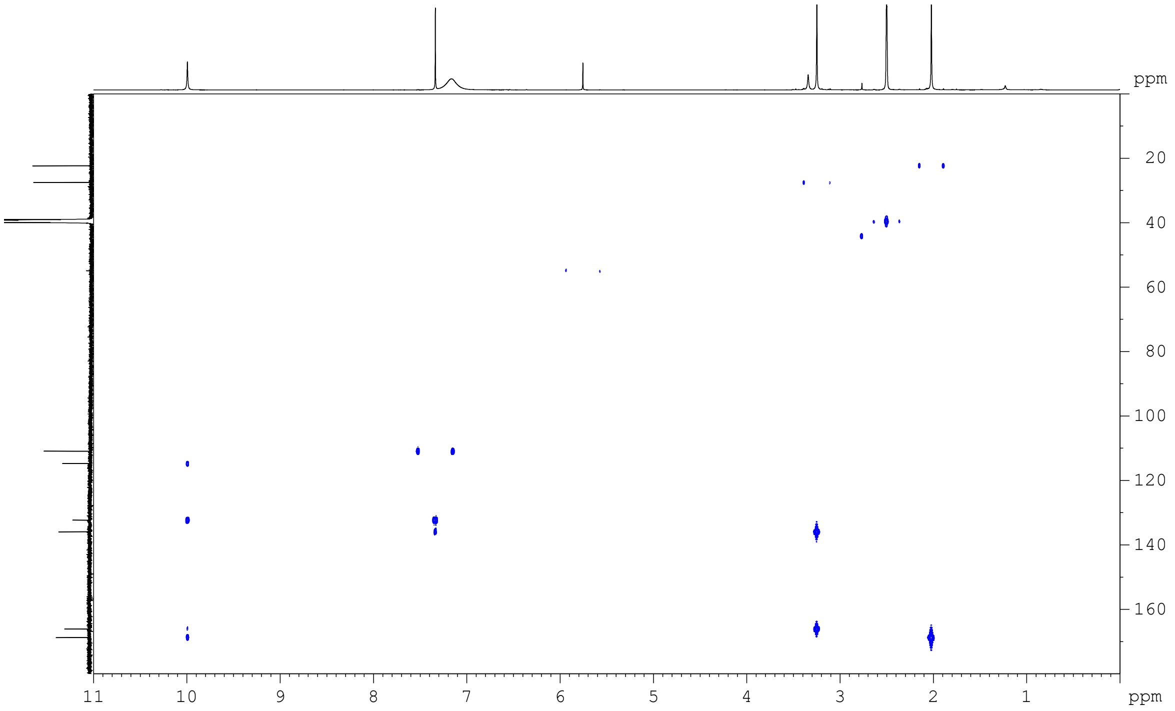

Figure S16. HMBC spectrum (500 MHz, DMSO-$_6$) of **3**

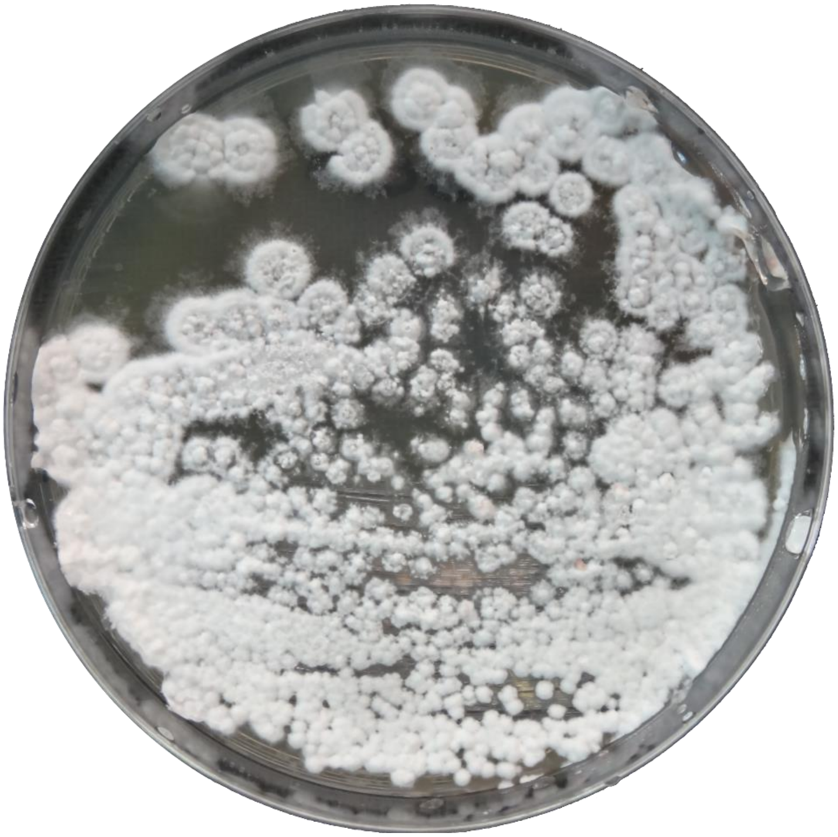

Figure S17. Colony characteristics of BTBU20218885

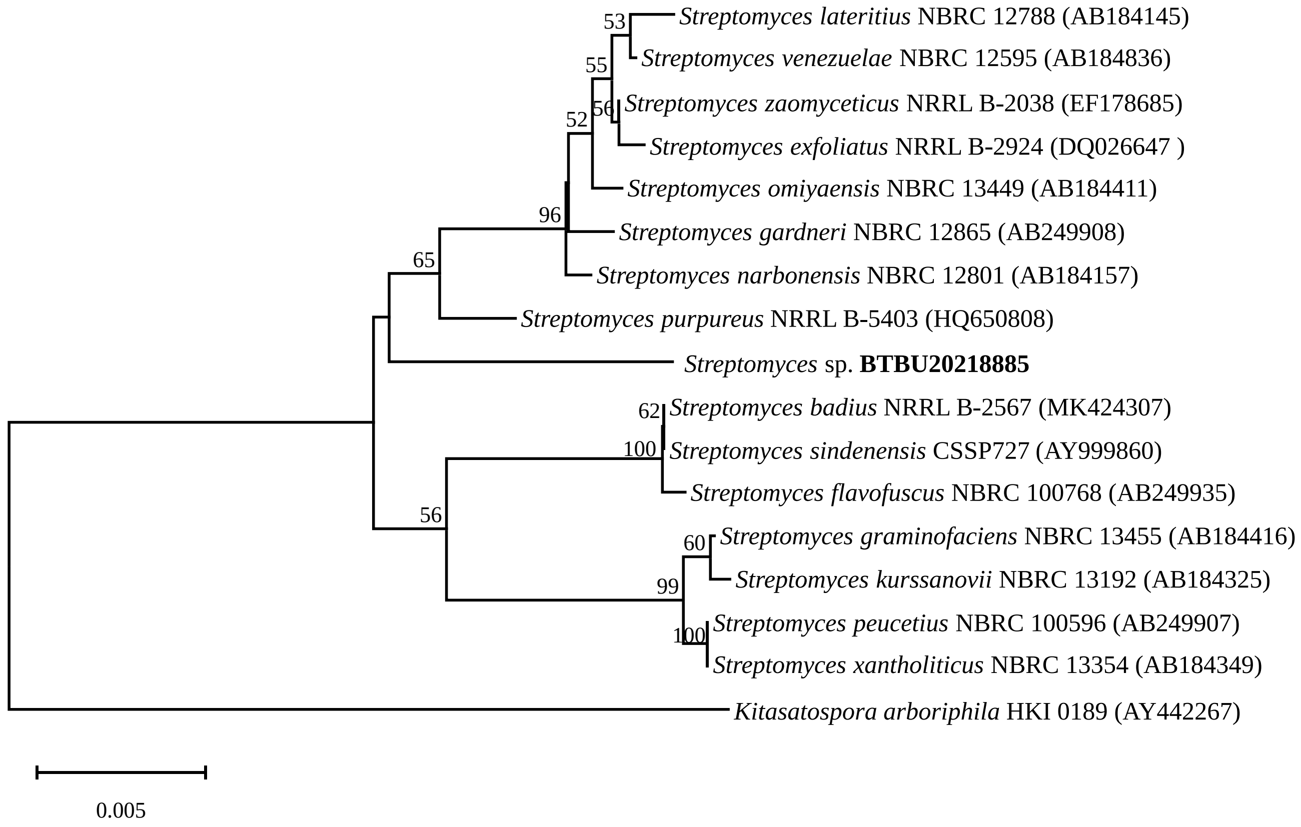

Figure S18. Neighbor-joining phylogenetic tree for *Streptomycetes* sp. strain BTBU20218885
